# The Evolving Landscape of Childhood Histiocytosis: A Decade of Discovery and Innovation

**DOI:** 10.3390/pediatric17030062

**Published:** 2025-05-23

**Authors:** Maurizio Aricò

**Affiliations:** Pediatrics, Azienda Sanitaria Locale, 65124 Pescara, Italy; maurizio.arico@asl.pe.it

## Abstract

Over the past decade, the field of childhood histiocytosis, particularly Langerhans cell histiocytosis (LCH), has undergone transformative changes. The integration of molecular genetics, targeted therapies, and refined diagnostic methodologies has revolutionized patient management and redefined disease classification. This editorial provides a comprehensive overview of the pivotal developments from 2015 to 2025, highlights ongoing challenges, and explores future directions in research and clinical care.

## 1. Introduction

Histiocytic disorders in children, notably Langerhans cell histiocytosis (LCH), have long represented a clinical and scientific challenge due to their heterogeneous presentations and unpredictable course. Historically considered an inflammatory or reactive condition, LCH is now recognized as a clonal neoplastic disorder rooted in myeloid progenitors. This paradigm shift over the past decade has catalyzed progress in diagnosis, risk stratification, and treatment.

## 2. Molecular Pathogenesis: A Defining Discovery

The discovery of recurrent somatic mutations in the MAPK (mitogen-activated protein kinase) signaling pathway, particularly BRAF V600E and MAP2K1 mutations, marked a watershed moment in understanding childhood LCH. Approximately 50–60% of patients harbor the BRAF V600E mutation, while an additional 20% exhibit MAP2K1 alterations [1,2]. These mutations result in the constitutive activation of the MAPK pathway, promoting the clonal proliferation of pathological dendritic cells.

The detection of BRAF V600E in circulating cell-free DNA has emerged as a powerful non-invasive biomarker, offering insights into disease burden and treatment response [3]. Moreover, the presence of this mutation in hematopoietic progenitor cells supports the notion that LCH arises early in myeloid differentiation. These findings have encouraged the exploration of hematopoietic stem cell’s contributions to disease persistence and relapse.

Recent studies have also suggested that MAPK pathway mutations are associated with distinct clinical phenotypes. For instance, BRAF V600E-positive patients are more likely to develop high-risk multisystem disease and experience the involvement of the central nervous system. These associations have implications for both prognosis and therapeutic decision-making.

## 3. Reclassifying Histiocytic Disorders

Molecular insights have driven a reclassification of histiocytic disorders. The revised classification by the Histiocyte Society and the WHO now categorizes LCH, Erdheim–Chester disease (ECD), and indeterminate cell histiocytosis under the “L” group, recognizing their shared MAPK-driven pathogenesis [4]. This classification reflects a more nuanced understanding of the disease continuum and encourages therapeutic cross-applicability.

This molecular reclassification has also spurred the development of novel disease models. Genetically engineered mouse models expressing BRAF V600E in CD11c+ dendritic cells recapitulate many features of human LCH, including CNS infiltration and cytokine dysregulation. These models have become invaluable for preclinical drug testing and mechanistic studies.

## 4. Clinical Heterogeneity and Diagnostic Advances

LCH displays a broad spectrum of clinical manifestations, from isolated bone lesions to life-threatening multisystem disease, with the involvement of risk organs (e.g., liver, spleen, or hematopoietic system). The skin, lungs, pituitary gland, and CNS are frequently involved, often leading to endocrinopathies, chronic pulmonary disease, and neurologic deficits.

MRI and PET-CT have become integral to disease staging and monitoring, allowing for better assessment of active lesions and responses to therapy. PET-CT, in particular, offers high sensitivity in detecting metabolically active lesions that may not be apparent on conventional imaging.

Moreover, liquid biopsies for BRAF V600E and other MAPK mutations provide a non-invasive means to track disease evolution and response. Serial cfDNA testing has been proposed as a prognostic tool, with early clearance correlating with treatment success.

Despite these tools, early diagnosis remains challenging. Delays can lead to irreversible damage, especially in the central nervous system (CNS) and endocrine system. Thus, increasing clinical awareness and improving access to molecular diagnostics are critical goals. The education of pediatricians, radiologists, and pathologists on the disease’s protean presentations is crucial for early detection.

## 5. Therapeutic Advancements and Targeted Therapy

Standard front-line therapy for multisystem LCH has long relied on vinblastine and prednisone [5,6,7,8]. However, relapse rates remain significant, and outcomes vary based on the involvement of risk organs. Second-line agents, including cytarabine and cladribine, have demonstrated efficacy in refractory cases. Revisited old agents, such as methotrexate and 6-mercaptopurine, are also being studied in combination regimens.

The introduction of targeted therapies has been a game changer. BRAF inhibitors such as vemurafenib and dabrafenib, and MEK inhibitors like trametinib, have shown high response rates in patients with refractory LCH harboring the relevant mutations [9]. Vemurafenib, for instance, leads to rapid clinical improvement and the radiological regression of lesions. Nonetheless, treatment cessation often leads to relapse, suggesting the need for prolonged therapy or combination regimens.

Furthermore, resistance to BRAF inhibitors has been observed, often mediated by secondary mutations or MAPK reactivation. Combination strategies targeting multiple nodes in the pathway or incorporating immunotherapy may overcome this limitation.

Pediatric-specific trials, such as those embedded in the LCH-IV protocol, are exploring optimal dosing, duration, and combination strategies. Importantly, long-term safety data, especially regarding growth and neurodevelopmental outcomes, are still maturing. These studies will inform future guidelines and ensure that therapies are tailored to the developing child.

## 6. Long-Term Complications and Quality of Life

Even with disease control, up to 50% of patients with multisystem LCH develop permanent sequelae, including diabetes insipidus, neurodegeneration, and growth failure [10]. Other complications include dental abnormalities, hearing loss, and orthopedic deformities.

CNS involvement, particularly in neurodegenerative LCH (ND-LCH), remains poorly understood and difficult to treat. Radiologically, ND-LCH is characterized by cerebellar and basal ganglia changes on MRI, which often precede clinical symptoms. Current approaches include IVIG, steroids, and MEK inhibitors, but with variable efficacy. There is an urgent need for biomarkers to predict and monitor CNS involvement.

Psychosocial challenges, including fatigue, cognitive impairment, and emotional distress, are increasingly recognized. Quality of life assessments reveal that even patients in remission report persistent symptoms that interfere with school performance and social engagement. Comprehensive survivorship care models are essential to address these multidimensional needs. Multidisciplinary follow-up, including from endocrinologists, neurologists, psychologists, and educational support teams, is recommended.

## 7. Emerging Concepts and Personalized Medicine

A shift toward personalized medicine is underway. Some propose stratifying patients based on molecular and inflammatory profiles—differentiating between malignancy-driven and inflammation-driven subtypes [11]. Such stratification could inform risk-adapted therapy and reduce overtreatment.

Additionally, immune profiling of lesional tissue has revealed a complex interplay between neoplastic Langerhans cells and the surrounding microenvironment. High levels of pro-inflammatory cytokines, including IL-17 and TNF-alpha, suggest that immunomodulatory therapies could be effective in selected patients.

In addition, immunotherapeutic strategies and vaccines targeting BRAF-mutant cells are under preclinical investigation. Advances in single-cell RNA sequencing and spatial transcriptomics promise to uncover novel targets and mechanisms of resistance. The integration of multi-omics data could lead to a new taxonomy of histiocytic disorders with therapeutic implications.

## 8. Challenges and Future Directions

Despite the progress we have outlined, several challenges remain:

1. Standardizing Molecular Testing: Access to and the standardization of molecular diagnostics is inconsistent globally. Many resource-limited settings lack the infrastructure for next-generation sequencing, hindering diagnosis and trial enrollment;
2. Relapse Management: Strategies for managing and preventing relapses, particularly after targeted therapy, require optimization. Longitudinal studies to define the optimal duration of maintenance therapy are needed;
3. Understanding CNS Disease: Mechanistic studies on ND-LCH are urgently required. Animal models and longitudinal neuroimaging studies may yield insights into pathogenesis and guide therapeutic development;
4. Long-Term Data: Prospective registries are essential to track long-term outcomes and treatment-related toxicities. Registries like Euro-Histio-Net and national databases in the US, France, and Japan are critical for capturing real-world data;
5. Equity in Care: Disparities in access to specialized care and molecular diagnostics must be addressed. Telemedicine, international collaboration, and educational initiatives can help to bridge these gaps.

Collaborative research efforts and clinical trial networks are vital. International initiatives such as the Histiocyte Society’s LCH-IV trial and the Euro-Histio-Net consortium play a critical role in advancing knowledge and care standards.

## 9. Conclusions

The last ten years have redefined childhood histiocytosis as a molecularly driven, clinically diverse neoplastic disorder. The integration of genomic insights with clinical management has improved outcomes and opened up new therapeutic avenues. However, to translate these advances into universally improved care, continued investment in research, access to diagnostics, and long-term follow-up infrastructure is essential. As we move forward, a precision medicine approach that balances efficacy, safety, and quality of life will be paramount.
